# Características clínicas y tiempos de atención en una unidad de dolor torácico del servicio de emergencias de un centro argentino

**DOI:** 10.47487/apcyccv.v4i2.293

**Published:** 2023-06-30

**Authors:** María Florencia Grande Ratti, Ignacio Martín Bluro, Fiorella Castillo, María Elena Zapiola, Ana Soledad Pedretti, Bernardo Martínez

**Affiliations:** 1 Instituto Universitario Hospital Italiano de Buenos Aires, Buenos Aires, Argentina. Instituto Universitario Hospital Italiano de Buenos Aires Buenos Aires Argentina; 2 Área de Investigación en Medicina Interna, Hospital Italiano de Buenos Aires, Buenos Aires, Argentina. Área de Investigación en Medicina Interna, Hospital Italiano de Buenos Aires Buenos Aires Argentina; 3 CONICET (Consejo Nacional de Investigaciones Científicas y Técnicas), Departamento de Medicina, Hospital Italiano de Buenos Aires, Buenos Aires, Argentina. CONICET (Consejo Nacional de Investigaciones Científicas y Técnicas) Departamento de Medicina Hospital Italiano de Buenos Aires Buenos Aires Argentina; 4 Central de Emergencias de Adultos, Hospital Italiano de Buenos Aires, Buenos Aires, Argentina. Central de Emergencias de Adultos Hospital Italiano de Buenos Aires Buenos Aires Argentina; 5 Servicio de Cardiología, Hospital Italiano de Buenos Aires, Buenos Aires, Argentina. Servicio de Cardiología Hospital Italiano de Buenos Aires Buenos Aires Argentina

**Keywords:** Urgencias Médicas, Atención de Enfermería, Manejo de Atención al Paciente, Dolor en el Pecho, Cardiología, Emergencies, Hospital, Nursing Care, Patient Care Management, Chest Pain, Cardiology

## Abstract

**Objetivos:**

. Reportar frecuencia de dolor precordial, describir características clínicas y tiempos de atención.

**Métodos:**

. Estudio descriptivo retrospectivo que incluyó consultas en la Unidad de Dolor Torácico del año 2021 en el servicio de urgencias de un hospital privado de Argentina.

**Resultados.:**

Hubo 1469 ingresos por dolor torácico, arrojando una frecuencia de 1,09% (IC95% 1,04-1,15). Fueron 52% hombres, con media de 62 años (DE ± 15); 48% hipertensión y 32% dislipemia. La mediana de tiempo a la realización del ECG inicial fue 4,3 min (RIC 2,5-7,5); y 26 min (RIC 14-46) hasta la evaluación médica. Se hospitalizaron 206 (14%) con mediana de 3 días, 76% ingresó a unidad cerrada, 9% requirió ventilación no invasiva/mecánica y la mortalidad intrahospitalaria fue de 2.9%. Los hospitalizados presentaron menor tiempo de demora a la atención médica (p<0,01), y mayor realización de estudios complementarios (p<0,01), sin diferencias en el tiempo al ECG (p=0,22).

**Conclusiones.:**

Los tiempos de atención estuvieron dentro de los estándares estipulados, siendo un importante indicador de calidad. Enfermería fue crucial, se ocuparon del triaje correcto, de la realización del ECG al ingreso, y de garantizar los cuidados hasta la evaluación médica.

## Abstract (en)

**Objectives:**

. To report the frequency of precordial pain, describe clinical characteristics, and care times.

**Methods:**

. Retrospective descriptive study that included consultations in the Chest Pain Unit in 2021 in the emergency department of a private hospital in Argentina.

**Results.:**

There were 1469 admissions for chest pain, yielding a frequency of 1.09% (95%CI 1.04-1.15). They were 52% men, mean age 62 years (SD ±15); 48% had hypertension and 32% dyslipidemia. The median time to initial ECG was 4.3 min (ICR 2.5-7.5); and 26 min (ICR 14-46) to medical evaluation. A total of 206 (14%) were hospitalized with a median of 3 days, 76% were admitted to a closed unit, 9% required non-invasive ventilation/mechanical ventilaction and in-hospital mortality was 2.9%. Those hospitalized presented shorter delay time to medical attention (p<0.01), and greater performance of complementary studies (p<0.01), with no differences in time to ECG (p=0.22).

**Conclusions.:**

Care times were within the stipulated standards, being an important indicator of quality. Nursing was crucial, taking care of the correct triage, ECG on admission, and guaranteeing care until medical evaluation.

## Introducción

La mortalidad cardiovascular sigue representando la causa más frecuente de pérdida de vidas en el mundo, donde el síndrome coronario agudo (SCA) es una de las causas subyacentes, que abarca angina inestable y/o SCA con y sin elevación del segmento ST ^1^. Todos estos cuadros, cuya presentación clínica más habitual es el dolor torácico, siguen representando un motivo de consulta frecuente en los ámbitos de urgencias ^2^. El SCA se asocia con elevada mortalidad sin el tratamiento apropiado ^3^; por ende, el diagnóstico correcto depende de su adecuada evaluación al ingreso.

Debido al volumen creciente de consultas no programadas y la limitada capacidad del sistema de salud para brindar respuesta ^4^, se han implementado estrategias operativas y protocolos asistenciales basadas en sistemas de triaje. Entre ellas, que el personal de enfermería entrenado permita iniciar el proceso de categorización del paciente, parece ser una estrategia prometedora ^5^. En ese contexto se creó la Unidad de Dolor Torácico (UDT) en el año 2014, el cual es un circuito estructurado para la atención de este subgrupo de pacientes, esto conllevó a agilizar el flujo, garantizar la atención precoz y la interconsulta (y/o evaluación temprana con especialistas en cardiología), basándose en la potencial gravedad de esta condición. El personal de enfermería cumple un papel primordial en este proceso asistencial, pues se ocupan de identificar las afecciones críticas a tiempo y así priorizar su atención por encima de aquellos que pueden esperar, con el fin de lograr brindar la atención necesaria, en el lugar correcto, el momento oportuno y con los recursos adecuados, de manera eficiente ^6^. Para poder asumir dicho rol reciben formación educativa en triaje de manera continua, debiendo recertificar cada 2 años por exigencia de documentación profesional.

Una vez que el paciente es asignado a la UDT, inmediatamente se realiza un electrocardiograma (ECG) analógico digitalizado ^7^, cuyo resultado es almacenado en la historia clínica electrónica (HCE), lo que permite brindar atención a distancia por parte de un especialista en cardiología (que puede entonces encontrarse físicamente en la unidad de cuidados coronarios, pero pudiendo proveer la lectura e interpretación de todas formas) ^8^. Luego, este proceso continúa mediante el juicio clínico de los médicos tratantes (habitualmente generalistas o clínicos), haciendo uso de las herramientas básicas (como antecedentes clínicos y examen físico), que cobran relevancia para enfrentar este desafío diagnóstico ^9^.

Existen dos tiempos que resultan clave para la UDT: el tiempo a la realización del primer ECG, y el tiempo de demora en la atención médica ^10^. Por lo que el presente estudio se propuso como objetivo principal reportar la frecuencia de dolor torácico en un servicio de emergencias, describir las características clínicas de estos pacientes, y reportar los tiempos de atención, siendo *proxys* de calidad y seguridad de atención sanitaria. Como objetivo secundario, exploramos los factores asociados a la hospitalización.

## Materiales y métodos

### Diseño y población de estudio

Estudio descriptivo retrospectivo que incluyó todas las consultas no programadas ocurridas durante el año 2021, en la Central de Emergencias de Adultos del Hospital Italiano de Buenos Aires, un centro de alta complejidad ubicado en la Ciudad Autónoma de Buenos Aires (Argentina), que cuenta con un servicio abierto 24 horas los 365 días del año, que atiende habitualmente un promedio de 350 consultas diarias. Está constituido por cuatro áreas para la atención, diferenciadas según la complejidad del paciente, la cual es definida por la condición al ingreso: Cuidados Críticos (Área A), Cuidados Intermedios (Área B), Consultorios de moderada complejidad (Área C), y consultorios de Demanda Espontánea o consultas de baja complejidad (Área D). Las áreas C y D corresponden a pacientes de menor complejidad, con motivos frecuentes en atención primaria, y representan el mayor flujo de los pacientes. Mientras que las áreas A y B representan el menor volumen, pero son los de mayor gravedad.

### Variables de estudio

Las variables de interés fueron provistas utilizando bases secundarias de alta calidad, provenientes de los registros en la HCE. La recogida de datos fue retrospectiva, se identificaron aquellos pacientes que fueron asignados a la UDT como primera área de ingreso, que como ya fue mencionado, fue definido por el responsable del triaje mediante personal de enfermería capacitado. Las variables administrativas recolectadas fueron: fecha y hora de llegada a servicio de urgencias, fecha y hora de atención médica -que permitió calcular tiempo de demora/espera-; fecha y hora de cierre de episodio -que permitió calcular tiempo global de estadía del paciente en urgencias-, y condición al alta (alta a domicilio, fallecimiento u hospitalización). Las variables de interés relacionadas al paciente fueron: edad, sexo, antecedentes y comorbilidades cardiovasculares, realización de ECG con fecha y hora -que permitió calcular tiempo a la realización-, así como otros estudios complementarios solicitados y/o tratamientos instaurados en urgencias.

En el subgrupo de pacientes que se hospitalizaron, se consignaron variables clínicas evolutivas adicionales tales como primer área de ingreso (unidad cerrada sí/no), pase a unidad cerrada en algún momento, requerimiento de ventilación no invasiva (VNI) o asistencia respiratoria mecánica (ARM), tiempo de estadía y muerte intrahospitalaria.

### Análisis estadístico

Se utilizó el análisis descriptivo, las variables numéricas se expresan como media y desvío estándar o mediana y rango intercuartílico, en tanto que las categóricas como números relativos y porcentajes, con sus respectivos intervalos de confianza de 95% (IC95%). Para explorar factores asociados a hospitalización se utilizó el análisis comparativo, mediante chi cuadrado o Fisher para variables dicotómicas, y T-test o Wilcoxon para variables numéricas (previa exploración de normalidad), considerando significancia estadística a valores de p<0,05. Se utilizó el *software* STATA versión 18.

### Consideraciones éticas

Este proyecto fue desarrollado cumpliendo los principios éticos acordes con las normas regulatorias de la investigación en salud humana a nivel nacional e internacional. El protocolo fue aprobado por el Comité de Ética institucional (CEPI#6412). Como se trataba de un estudio observacional y retrospectivo no se requirió de la firma de consentimiento informado de los participantes.

## Resultados

**Figura 1:**
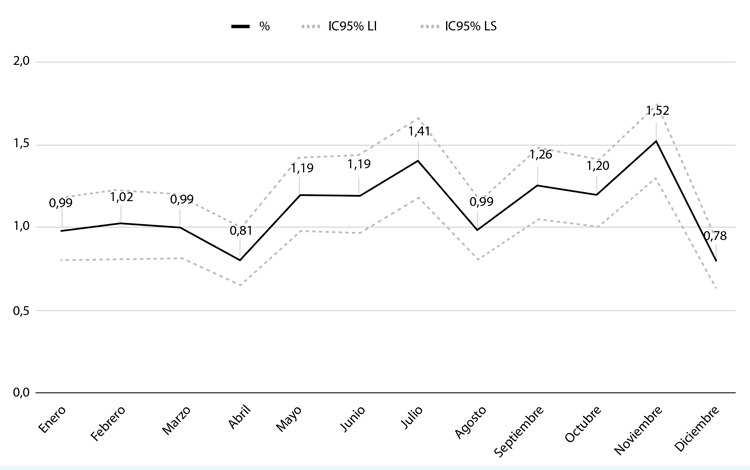
Frecuencia de consultas de Unidad de Dolor Torácico en servicio de urgencias, durante año 2021, estratificado por mes.

Durante el período del estudio ocurrieron 133607 consultas totales, y solo 1469 correspondieron a los ingresados en UDT, arrojando una prevalencia global del 1,09% (IC95% 1,04-1,15). La Figura 1 muestra estabilidad de casos de dolor torácico al estratificar por mes, con un promedio de 122 al mes, con el valor más bajo en diciembre (0,78%; IC95% 0,64-0,93) y el valor más alto en noviembre (1,52%; IC95% 1,31-1,74).

Como puede observarse en la Tabla 1, los pacientes de UDT presentaron una edad media de 62 años y 52% fueron de sexo masculino. Las comorbilidades cardiovasculares preexistentes más frecuentes fueron hipertensión arterial (48%) y dislipidemia (32%).

**Tabla 1:** Características clínicas y demográficas de los pacientes en Unidad de Dolor Torácico durante el 2021

Características	n: 1469	Hospitalizados (n: 206)	No hospitalizados (n: 1263)	p-valor
Sociodemográficas				
Edad, en años *	62,92 (15,91)	69,61 (13,25)	61,83 (16,04)	0,001
Sexo masculino	52,69% (774)	64,56% (133)	50,75% (641)	0,001
Antecedentes cardiovasculares				
Hipertensión arterial	48,40% (711)	61,17% (126)	46,32% (585)	0,001
Dislipemia	31,99% (470)	39,81% (82)	30,72% (388)	0,010
Sobrepeso	19,54% (287)	19,90% (41)	19,48% (246)	0,886
Tabaquismo	19,06% (280)	22,33% (46)	18,53% (234)	0,198
Diabetes	11,57% (170)	17,48% (36)	10,61% (134)	0,004
Enfermedad renal crónica	3,68% (54)	6,31% (13)	3,25% (41)	0,030
Sedentarismo	1,09% (16)	1,94% (4)	0,95% (12)	0,204
Hospitalización en unidad coronaria durante el año previo	9,66% (142)	12,14% (25)	9,26% (117)	0,196
Tiempos de atención				
Tiempo al electrocardiograma (entre llegada hasta realización de este), en minutos **	4,33 (2,48-7,5)	3,8 (2,2-7,1)	4,4 (2,5-7,6)	0,216
Tiempo de demora/espera (entre llegada hasta atención por médico), en minutos **	25,98 (13,71-45,63)	18,18 (11,4-29,5)	27,19 (14,86-48,75)	0,001
Tiempo atención (aquel transcurrido entre atendido por médico hasta cierre del episodio de urgencias), en minutos **	135,38 (63,51-234,78)	100,7 (39,2-170,1)	138,9 (72,6-245,1)	0,001
Tiempo global del paciente (entre llegada hasta cierre del episodio de urgencias), en horas **	2,81 (1,66-4,48)	2,1 (0,9-3,3)	2,9 (1,8-4,7)	0,001
Tiempo de evolución de Cardiología (entre llegada hasta evolución médica por especialista), en minutos **	137,1 (63,9-206,9)	74,6 (25,2-155,4)	148,6 (90,5-229,1)	0,001

*Media (desvío estándar)

**Mediana (PC25-PC75)

La mediana de tiempo a la realización del ECG fue de 4,33 min (RIC 2,48-7,5), mientras la mediana de tiempo de demora en la atención médica fue de 25,98 min (RIC 13,7-45,6). Solo 206 sujetos finalizaron esta consulta no programada con una hospitalización (14%), y hubo un solo caso de fallecimiento en urgencias (que obitó sin hospitalizarse). Se trató de un hombre de 41 años que ingresó por dolor precordial, en el que se constató supradesnivel del ST en cara lateral alta (D1 y aVL), con especularidad inferior (D2, D3, aVF). Evolucionó con intensificación del dolor, fibrilación ventricular y paro cardiorrespiratorio.

**Figura 2:**
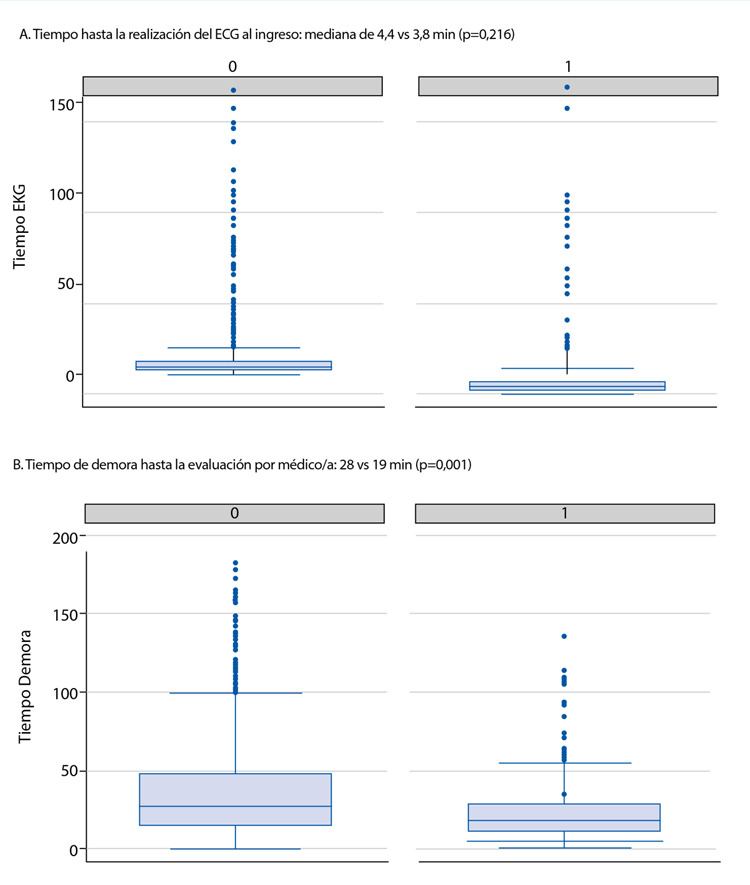
Tiempos de atención, estratificados por hospitalización.

Tal como muestra la Figura 2, al estratificar a los pacientes por su condición al cierre de la epicrisis, según hospitalización, no hubo diferencias significativas en los tiempos a la realización del primer ECG (mediana 3,8 vs 4,4 min respectivamente; p=0,216), pero sí en la demora en la atención médica (mediana 18 vs. 27 min; p=0,001).

1: hospitalizados; 0 : no hospitalizados.

En cuanto a la realización de estudios complementarios, se observaron diferencias en la frecuencia de uso de laboratorio, radiografía de tórax y ecocardiograma. Sin embargo, fue similar la frecuencia del registro del ECG inicial. En cuanto a los resultados de laboratorio, los pacientes hospitalizados presentaron mayores valores de troponina, de creatininemia y de BNP tal como puede observarse en la Tabla 2.

**Tabla 2:** Características de laboratorio y tratamiento de los pacientes en Unidad de Dolor Torácico durante el 2021

Características	n: 1469	Hospitalizados (n: 206)	No hospitalizados (n: 1263)	p-valor
Estudios complementarios y tratamiento en urgencias				
Electrocardiograma	91,15% (1339)	92,72% (191)	90,89% (1148)	0,393
Laboratorio	70,80% (1040)	95,63% (197)	66,75% (843)	0,001
Troponina	59,29% (871)			
Radiografía de tórax	29,34% (431)	49,51% (102)	26,05% (329)	0,001
Ecocardiograma	7,69% (113)	18,93% (39)	5,86% (74)	0,001
Evolución de Cardiología	25,32% (372)	43,69% (90)	22,33% (282)	0,001
Aspirina	5,03% (74)	33,01% (68)	0,48% (6)	0,001
Beta bloqueantes	2,04% (30)	3,88% (8)	1,74% (22)	0,044
Drogas inotrópicas	0,34% (5)	1,94% (4)	0,08% (1)	0,001
Nitratos/Nitritos	4,69% (69)	25,24% (52)	1,35% (17)	0,001
Hospitalización	14,02% (206)	N/A	N/A	N/A
Resultados de laboratorio				
Hto, frecuencia (%)	67,32% (989)	95,14% (196)	62,78% (793)	0,001
Hto, valor **		41,35 (37,1-44,0)	39,8 (37,2-42,3)	0,002
CPK, %	22,12% (325)	37,86% (78)	19,55% (247)	0,001
CPK, U/mL**		88,50 (63-140)	83,00 (60-123)	0,095
Troponina, frecuencia (%)	59,29% (871)	86,40% (178)	54,86% (693)	0,001
TnT, número de mediciones		1 (1-1)	1 (1-1)	0,712
> de 1 medición de TnT	21,69% (189/871)	23,03% (41/178)	21,35% (148/693)	0,628
TnT, primer valor en pg/mL**		19,7 (9,6-66,2)	8,1 (5,5-12,4)	0,001
Creatininemia, frecuencia (%)	66,71% (980)	93,68% (193)	62,31% (787)	0,001
Creatininemia, valor en mg/dL **		0,93 (0,78-1,13)	0,88 (0,73-1,04)	0,002
BNP, frecuencia (%)	8,30% (122)	27,18% (56)	5,22% (66)	0,001
BNP, valor en pg/mL**		994,8 (181,8-3344,5)	415,3 (123,8-1690)	0,044

*Media (desvío estándar)

**Mediana (PC25-PC75)

De los 206 pacientes hospitalizados, la mayoría (76,70%) ingresó a una unidad cerrada como primer área de ingreso (definida como unidad coronaria, unidad de terapia intermedia y/o unidad de terapia intensiva), y permanecieron una mediana de 3 días (RIC 2-5). Sin embargo, de los restantes 48 sujetos que ingresaron a sala general como primera área de ingreso, hubo una intercurrencia del 62,5% con requerimiento y pase a unidad cerrada. En lo que respecta a la evolución clínica, 19 pacientes (9,22%) requirieron ventilación no invasiva y/o asistencia respiratoria mecánica, y la mortalidad intrahospitalaria resultó 2,91%.

## Discusión

El dolor precordial constituyó el 1% de todas las consultas no programadas a lo largo del año 2021, y los tiempos clave del proceso asistencial estuvieron dentro de los estándares estipulados por las recomendaciones de las guías de práctica clínica. Nuestros hallazgos (mediana de 4 min a la realización del primer ECG; y 26 min de demora para la evaluación médica) son similares a estudios previos que refieren que el tiempo de asistencia para SCA oscila entre 20-30 min ^11^, con el primer ECG en una media de 20-25 min ^12^^,^^13^, aunque estos dos últimos reportaron media.

Todos nuestros hallazgos documentan que la disponibilidad de un sistema de triaje, y la atención diferencial por parte de enfermería, garantiza la atención temprana, y debería considerarse como un indicador de calidad relevante de la relación riesgo-eficiencia, lo que resulta consistente con la literatura ^14^^,^^15^. Estos profesionales han demostrado poseer un rol crucial en el cuidado del paciente crítico en urgencias, particularmente en la UDT, no solo por la alta capacidad para llevar a cabo el triaje correcto (categorización que facilita brindar la atención necesaria, en el lugar correcto, el momento oportuno, con los recursos adecuados, y de manera eficiente), sino por la realización y la evaluación del ECG al ingreso en forma temprana, y el hecho de garantizar los cuidados iniciales de atención hasta la evaluación médica.

El tiempo de demora a la atención médica fue menor en pacientes hospitalizados, en comparación con aquellos no hospitalizados, lo que probablemente sugiere que, ante resultados de ECG normales y la posibilidad de revisión de HCE con datos confiables para riesgo cardiovascular pre-prueba, son atendidos más rápido aquellos pacientes con mayor probabilidad de enfermedad coronaria. En consecuencia, probablemente se produzca una subestratificación de las prioridades de atención luego de realizar el ECG. En forma consistente, la presencia de comorbilidades se asoció con una mayor chance de hospitalización, lo que podría explicarse por los casos potencialmente más graves. El SCA es considerado una patología tiempo-dependiente; por ende, un posible caso debería ser evaluado y tratado rápidamente. Estos hallazgos sugieren una reevaluación del riesgo muy precoz, realizada por especialistas en cardiología, y eventualmente acompañada de curva de enzimas cardíacas, que se llevó a cabo casi en una cuarta parte de los sujetos (22% tuvo más de una medición de troponinas).

Cabe mencionar que más que en cualquier otro sector del hospital, el concepto de flujo de trabajo y el mantenimiento de la eficiencia es fundamental para el éxito de la práctica de la medicina en el ámbito de urgencias; sin embargo, existe cierta discrepancia entre el acto médico y el registro de este en la HCE ^16^. Entonces, sigue ocurriendo que se atiende al paciente en primera instancia y luego registra a destiempo. Algo similar podría ocurrir con la evolución por cardiología (solo registrada en un 25%), y con una mediana de 2 horas desde el ingreso del paciente.

La frecuencia del 1% de consultas por dolor torácico resultó baja en comparación con otros estudios epidemiológicos que utilizan un sistema de triaje similar, como Estados Unidos (5%) ^17^ o Noruega (11%) ^18^. Tal vez, podría explicarse por la ventana temporal del estudio, pudiendo estar aún teñida por la pandemia COVID19, coincidiendo con la segunda ola (junio 2021) y tercera ola (diciembre 2021), que hace predominar otros motivos de consulta relacionados como fiebre (5,1%), odinofagia (4,7%), y dolor abdominal (2,6%) ^19^.

Pese a la existencia de protocolos diagnósticos para estratificar el riesgo de estos pacientes, (incluso validados) que se han convertido en componentes centrales de las pautas de práctica actuales ^20^ el miedo a pasar por alto el SCA sigue siendo un fuerte motivador para que los médicos realicen más estudios complementarios a sus pacientes en los servicios de urgencias ^21^. Indefectiblemente, los resultados demuestran que se realizaron con mayor frecuencia estudios de laboratorio radiografía de tórax y ecocardiograma en los pacientes que se hospitalizaron. Esto podría interpretarse por un sesgo de información relacionado a los casos más graves; es decir, el mayor riesgo cardiovascular basal de estos sujetos. En consistencia con esto, los factores asociados a la hospitalización (edad, sexo masculino y preexistencias cardiovasculares) coincidieron con los descritos en la bibliografía ^22^. Los profesionales pueden sentirse presionados por las fuerzas opuestas de la realidad clínica y la necesidad de publicar indicadores clave de desempeño exitosos en un entorno de demandas crecientes y contención de costos ^23^.

Cabe mencionar algunas limitaciones. En primer lugar, es un estudio unicéntrico, lo que atenta contra la validez externa. En segundo lugar, se trata de un análisis retrospectivo y no ha sido factible recolectar otras variables de interés por la naturaleza de la captura del dato del estudio. En tercer lugar, dado que los pacientes suelen presentar múltiples problemas o motivos de consulta, puede ser controversial la forma de recolección de datos (restringida a pacientes ingresados en UDT), es decir, otros pacientes con dolor torácico o de origen coronario pueden haber sido excluidos por mala clasificación al triaje (falsos negativos: asignados a otras áreas originalmente, pero luego reclasificados a UDT). En contrapartida, como fortaleza, no hemos encontrado estudios previos que hayan descrito este tópico en un servicio de urgencias local ni regional, por lo que estos datos cobran gran relevancia para la gestión. Otro aspecto positivo fue el muestreo consecutivo de todas las consultas, lo que evita el sesgo de selección relacionado a un muestreo.

En conclusión, la mayoría de los pacientes con dolor torácico son atendidos precozmente. La evaluación por la potencial gravedad de esta condición incluye el juicio y las herramientas clínicas básicas (electrocardiograma, antecedentes cardiovasculares, y examen físico). La función de enfermería en este proceso es primordial, no solo se ocupan del triaje correcto en UDT, sino de la realización de ECG al ingreso, y de los cuidados iniciales del paciente. La mayoría se realizó laboratorio (70%) y marcadores cardíacos con troponina (60%). Sin embargo, se hospitalizaron pocos (14%), probablemente aquellos de mayor riesgo. Serán necesarios futuros estudios que exploren las características de los pacientes que se internaron sin medición de troponina (probablemente los más graves), los costos sanitarios asociados (ejemplo: curva de troponina, mortalidad), y/o la evolución clínica de los hospitalizados posexternación ^24^.
